# Exploring the attitudes of non- psychiatric healthcare workers towards patients with alcohol use disorder in a tertiary hospital

**DOI:** 10.1192/j.eurpsy.2023.820

**Published:** 2023-07-19

**Authors:** H. T. Tan

**Affiliations:** Psychiatry, Institute of mental health, Singapore

## Abstract

**Introduction:**

There are serious adverse effects on the physical and mental wellbeing of patients with alcohol use disorders.

It is important to screen and provide brief intervention for these group of patients during their inpatient admission.

Prompt identification and treatment of patients with alcohol use disorders are contingent on the attitudes of healthcare workers towards them.

Non-psychiatric doctors and nurses might respond inadequately due to negative attitudes and beliefs.

**Objectives:**

We examined the attitudes of non-psychiatric workers in the medical and surgical wards.

**Methods:**

The Alcohol & Alcohol-Problems Perceptions Questionnaire (AAPPQ) was administered to 128 doctors and 785 nurses from the medical and surgical disciplines in a tertiary hospital.

**Results:**

75.5% of doctors and 51.9% of nurses endorsed the domain of role legitimacy in the AAPPQ.

However both groups reported low-levels of role-adequacy (combined: 41.2%), role-support (combined: 36.9%), motivation (combined: 36.5%), task-specific self-esteem (combined: 25.1) and work satisfaction (combined: 20.5%) in the AAPPQ.

**Conclusions:**

While non-psychiatric healthcare workers acknowledged the importance to initiating intervention for patients with alcohol-use disorder in daily work, there were low levels of therapeutic commitments towards patients with problematic alcohol-use.

It is vital to introduce in-house programmes to educate, empower and emphasise the importance of therapeutic contact with patients for alcohol intervention.

**Disclosure of Interest:**

None Declared

